# Comparison of Flunixin Meglumine, Meloxicam and Ketoprofen on Mild Visceral Post-Operative Pain in Horses

**DOI:** 10.3390/ani12040526

**Published:** 2022-02-21

**Authors:** Louise C. Lemonnier, Chantal Thorin, Antoine Meurice, Alice Dubus, Gwenola Touzot-Jourde, Anne Couroucé, Aurélia A. Leroux

**Affiliations:** 1 Department of Clinical Sciences, Equine Veterinary Teaching Hospital (CISCO), Oniris, Route de Gachet, F-44000 Nantes, France; antoine.meurice@oniris-nantes.fr (A.M.); gwenola.touzot-jourde@oniris-nantes.fr (G.T.-J.); anne.courouce@oniris-nantes.fr (A.C.); aurelia.leroux@oniris-nantes.fr (A.A.L.); 2 Nutrition PhysioPathologie et Pharmacologie, University of Nantes, Oniris, F-44000 Nantes, France; chantal.thorin@oniris-nantes.fr; 3 Clinique Vétérinaire de Bel Air, Allée Marcel Doret, F-41000 Blois, France; alice.dubus@orange.fr; 4 UMR Inserm U1229–Regenerative Medecine and Skeleton, Department of Veterinary Clinical Sciences, University of Nantes, Oniris, F-44000 Nantes, France; 5 Biotargen, Normandie University, Unicaen, F-14000 Caen, France; 6 l’Institut du Thorax, Université de Nantes, CHU Nantes, CNRS, INSERM, F-44000 Nantes, France

**Keywords:** analgesia, anti-inflammatory, castration, pain score, equine

## Abstract

**Simple Summary:**

Pain management following surgical intervention is key. In horses, several anti-inflammatories (flunixin meglumine, meloxicam and ketoprofen) are available for the management of pain following castration. However, their analgesic efficacy remains unclear for mild visceral pain. The aim of this study was to compare the analgesic efficacy of the above-mentioned anti-inflammatory drugs, following a simple surgery (inguinal castration). Horses were administered a randomly assigned anti-inflammatory drug before and after surgery. A pain score was recorded using a previously described pain assessment scale (PASPAS) before administration, and after the first and second administrations by a senior clinician and a veterinary student. Thirty horses were evaluated, and there was no significant effect of the drug administered on the pain score. Horse welfare was not compromised regardless of drug assigned. Horses showed mild pain post-operatively, which decreased significantly within 24 h. Pain scores were significantly different when assessed by a veterinary student and a senior clinician. The authors found that the anti-inflammatory drug studied provided a similar level of analgesia for the management of mild visceral pain in horses, but that the pain scale used is not suitable for junior evaluators or, by extension, owners.

**Abstract:**

The analgesic efficacy of meloxicam and ketoprofen against equine visceral pain is unclear. The aim of this study was to compare the analgesic efficacy of meloxicam (M) and ketoprofen (K) to flunixin meglumine (F) following inguinal castration. Horses undergoing inguinal castration under general anesthesia were randomly assigned F (1.1 mg/kg), M (0.6 mg/kg) or K (2.2 mg/kg) intravenously two hours pre-operatively and 24 h later. A pain score (out of 31) was recorded blindly by a senior clinician and veterinary student before NSAIDs administration (T_0_), and after the first (T_1_) and second (T_2_) administrations, using a modified post-abdominal surgery pain assessment scale (PASPAS). Pain was classified as mild (score ≤ 7), moderate (score = 8–14) or severe (score > 14). Thirty horses (12 F, 10 M, 8 K) aged 6.2 ± 4.9 years, mostly warmbloods, were included. Horse welfare was not compromised regardless of the drug assigned. There was no statistically significant effect of NSAIDs on pain score. Mean pain scores were significantly higher at T_1_ than T_0_ for each NSAID (F: 5.08 ± 2.50 vs. 1.58 ± 1.38 (*p* < 0.001); M: 4.60 ± 2.32 vs. 1.10 ± 1.20 (*p* < 0.001); K: 5.25 ± 1.39 vs. 1.50 ± 1.51 (*p* < 0.0001)) and lower at T_2_ than T_1_ for F (2.92 ± 2.423 vs. 5.08 ± 2.50 (*p* < 0.001)) and M (2.90 ± 1.37 vs. 4.60 ± 2.32 (*p* < 0.0325)). At T_1_, senior pain scores were significantly different than for junior (5.56 ± 0.54 vs. 3.22 ± 0.62, *p* = 0.005). This study indicates that meloxicam and ketoprofen provide a similar level of analgesia to flunixin meglumine for the management of mild visceral pain in horses. PASPAS is not reliable for junior evaluators.

## 1. Introduction

Non-steroidal anti-inflammatory drugs (NSAIDs) are routinely used in horses for their anti-inflammatory and analgesic effects, with flunixin meglumine being reported as the drug of choice for the management of visceral pain in horses [1]. In addition, recent guidelines regarding pain management in horses state that horses should receive NSAIDs prior to surgery, and that analgesia should be continued for 3 days following castration [2]. However, non-cyclooxygenase (COX)-specific NSAIDs like flunixin meglumine are known to have significant adverse effects, such as, but not limited to, gastrointestinal ulceration and impaired renal function. Other NSAIDs, reported to be more selective, such as meloxicam or ketoprofen, appear to cause less side effects [3,4,5]. Even though meloxicam and ketoprofen are recommended for analgesia in cases of colic based on marketing authorizations, their analgesic efficacy remains unclear for mild visceral pain.

Assessment of pain in horses is challenging, and there is no gold standard model described for pain assessment in horses. Numerous composite pain scales have been developed and compared, depending on the type of pain evaluated (musculoskeletal, visceral, etc.). Castration is a commonly performed surgical procedure in horses, and it has been associated with some degree of pain that can persist for several days. Inguinal castration has been used as a model of pain in previous studies and was considered a suitable model in this study [6,7]. Animal welfare is central in post-operative care. However, there is currently no gold standard for the assessment of pain in horses, thus, there is a need for evidence-informed approaches in order to optimize equine welfare [8]. Furthermore, the reliability of a pain score when assessed by a veterinary student has been evaluated in dogs and in laminitic horses, with significant differences found when compared to scores obtained from experienced anesthesiologists or experienced veterinarians [9,10]. However, such comparisons have not been done for the assessment of visceral pain.

The aims of this study were to: (1) assess the inter-observer reliability of a composite pain score (CPS) between an experienced veterinarian and a fourth-year veterinary student, and (2) compare the analgesic effects of flunixin, meloxicam and ketoprofen on visceral pain following inguinal castration, with the use of the same CPS.

## 2. Materials and Methods

### 2.1. Study Sample

Client-owned horses admitted to the Equine Veterinary Teaching Hospital of Oniris (CISCO) for inguinal castration from February 2019 to June 2021 were included. Owners were required to sign an informed consent form at admission as part of the pre-operative paperwork. Ethical review and approval were waived as per national ethical requirements, and horse welfare was not compromised regardless of drug administered. Horses were considered healthy based on a pre-operative examination, which included a physical examination, rebreathing pulmonary auscultation, electrocardiogram (ECG) at rest and routine hemato-biochemistry. Cases of abdominal cryptorchidism, coelioscopic castration or scrotal castration were excluded, whereas cases of inguinal cryptorchidism were included. Horses were included in the study upon surgeon and anesthetist’s acceptance.

Horses were housed in individual stalls with shavings and free access to water. Free access to hay was given until 8 h prior to surgery. All horses were tranquilized with acepromazine (0.02 mg/kg bwt IV, Calmivet^®^, Vetoquinol, Lure, France) at the time of jugular catheter placement, one hour before sedation with romifidine (0.08 mg/kg bwt IV, Sedivet^®^, Boehringer Ingelheim Animal Health, Lyon, France). Anaesthesia was induced with diazepam (0.05 mg/kg bwt IV, Valium^®^, Atnahs Pharma Netherlands B.V., Kobenhavn, Denmark) and ketamine (2.2 mg/kg bwt IV, Imalgene^®^ 1000, Boehringer Ingelheim Animal Health, Lyon, France), then placed in dorsal recumbency and maintained by inhalational anesthesia with isoflurane (Vetflurane^®^, Virbac, Carros, France). All horses received a bolus of morphine (0.1 mg/kg bwt IV, Morphine^®^, Cooperation Pharmaceutique Française, Melun, France) at the beginning of surgery and local anesthesia was carried out by intratesticular injection of 10 mL of lidocaine 2% per testicle (Lurocaine^®^, Vetoquinol, Lure, France), as recommended [2,11]. Inguinal castration was conducted by one of the four specialists and/or one of the two residents of the European College of Veterinary Surgery (ECVS). Prophylactic antibiotherapy (procain benzylpenicillin, 22,000 UI/kg bwt IM q12h, Depocilline^®^, Intervet, Beaucouzé, France) was initiated pre-operatively and maintained for 48 h. All horses received anti-edematous medication, first intravenously following surgery (dexamethasone 0.01 mg/kg bwt, hydrochlorothiazide 1.0 mg/kg bwt IV, Diurizone^®^, Vetoquinol, Lure, France), then orally (dexamethasone 0.01 mg/kg bwt, trichlormethiazide 0.5 mg/kg bwt PO, Oedex^®^, Dopharma, Vair sur Loire, France) the next day in order to prevent post-operative edema formation [12].

Horses were randomly allocated an NSAID by the anesthesiologist via a randomized matrix and blinded drawing: flunixin meglumine (1.1 mg/kg bwt IV, Antalzen^®^, Laboratorios Calier, Barcelona, Spain), meloxicam (0.6 mg/kg bwt IV, Rheumocam^®^, Audevard, Clichy, France) or ketoprofen (2.2 mg/kg bwt IV, Ketofen^®^, Ceva Sante Animale, Libourne, France). NSAID administration was given pre-operatively within one hour of induction, and 24 h later.

### 2.2. Pain Score

In order to assess post-operative pain, horses were evaluated using a post-abdominal surgery pain assessment scale (PASPAS) from Graubner et al., adapted to be used as a castration model [13]. The parameters assessed included subjective evaluation of pain, heart rate, respiratory rate, postural and interactive behavior, appetite, signs of colic and response to thoracolumbar palpation. An estimation of inflammation was characterized by the degree of incisional edema instead of palpation of the surgical site, in order to prevent contamination (Table 1). A score out of 31 was obtained, and pain was graded as mild (score 0–7), moderate (score 8–14) or severe (score > 14).

### 2.3. Study 1—Variability of Pain Scores Based on Evaluator’s Experience

In order to assess the accuracy of the pain score when performed by a veterinary student, horses were simultaneously evaluated by a fourth-year veterinary student (AD) hereafter named Junior (Jr), in addition to the experienced internist (AL) hereafter named Senior (Sr). Both evaluators were unaware of the treatment allocation, assessed horses separately within 30 min to 3 h of each other, and did not share the obtained pain scores with each other.

### 2.4. Study 2—Analgesic Effect of NSAIDs According to the Pain Score

**Figure 1:**
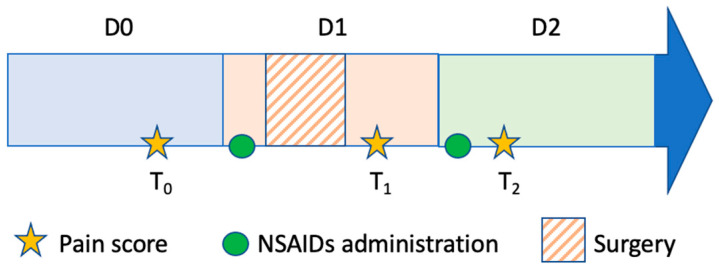
Study protocol. NSAID was administered 1 h before surgery and 24 h later. Pain scores were assessed before NSAID administration (T_0_), after recovery from anesthesia (T_1_) and after the second NSAID administration (T_2_).

In order to assess the analgesic effect of each NSAID, pain scores were recorded at 3 time points: the day before surgery and drug administration (T_0_), on the day of surgery after complete recovery from anesthesia (T_1_, 9–12.5 h after drug administration) and on the next day (T_2_, 1–9.5 h after drug administration) (Figure 1**)**. Horses were evaluated by a specialist (AL) or a second-year resident (LL) of the European College of Equine Internal Medicine (ECEIM). Evaluators were blinded to the treatment administered, and the same evaluator assessed the same horse once at each time point. Behavioral parameters and respiartory rate were first observed from outside the stall to reduce the impact of the evaluator’s presence. Heart rate was then assessed by auscultation, incisional oedema was assessed visually, and superficial palpation of the thoracolumbar area was performed last to assess local pain.

### 2.5. Statistical Analysis

Statistical analyses were performed using R (Version 4.0.5, packages: readl_nime, multcomp, ggplot2, plyr—©2009–2016) with significance set at *p* ≤ 0.05. Horses were randomly assigned to a NSAID group and a Kruskal Wallis test was used to assess age homogeneity between groups. Pain scores obtained at T_0_ were substracted from scores obtained at T_1_ and T_2_ to minimize the impact of individual variations. Negative results were corrected to 0 and corrected pain scores were used for statistical analysis. To take into account the repeated measurement design, linear mixed-effect models with random effects on horse factor were used to evaluate: (i) the effect of NSAIDs and time on pain score, (ii) the effect of time span from drug administration to pain score assessment, and (iii) the reliability of the pain score between a senior and a junior evaluator. The independence and normal distribution of residuals and random effects was checked for each model (data not shown). Multiple comparisons of means were also performed with post-hoc Tukey tests. The effect of the presence of inguinal cryptorchidism on NSAIDs distribution was assessed using Fisher’s exact test, and the effect on pain scores was assessed using mixed models and Tukey tests. In the descriptive approach, data were expressed as the mean and standard error. Results of mixed models were expressed as the estimate and standard deviation.

## 3. Results

### 3.1. Study Sample

Forty-three horses were initially included in the study. Seven horses were excluded because of a change of NSAID to phenylbutazone (2.2 mg/kg bwt PO, Equipalazone^®^, Dechra Regulatory, Ae Bladel, The Netherlands) on day 2. In those cases, the jugular catheter was removed directly following recovery from anesthesia for various reasons at the surgeon’s or anesthetist’s discretion (accidental removal during recovery, inflamed jugular vein, patient at risk of complications), and oral medication was then preferred.

All horses recovered uneventfully from general anesthesia, and only mild post-operative complications were observed, including isolated colic episodes (*n* = 1), colitis (*n* = 1), hyperthermia (*n* = 1) and surgical site hematoma (*n* = 2).

### 3.2. Study 1—Variability of Pain Scores Based on Evaluator’s Experience

**Figure 2:**
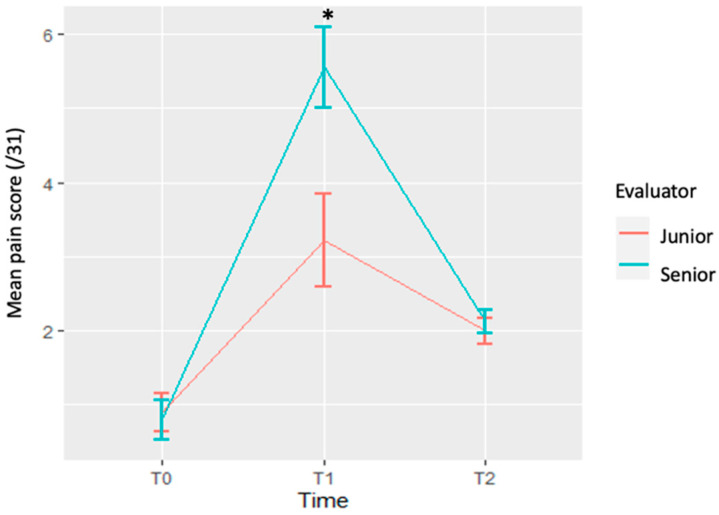
Comparison of pain scores assessed from a specialist (senior) and a veterinary student (junior). Data reported as the mean and SEM. * Significant difference between scores from senior and junior (*p* ≤ 0.05).

Ten horses were evaluated by both an experienced veterinarian (AL) and a fourth-year veterinary student (AD). The mean age was 6.6 years (range 1.0–20 years) and the represented breeds included mixed-breed leisure horses (5/10), French Warmbloods (2/10), Lusitanians (2/10) and one Thoroughbred. Horses were included independently of the administered NSAID, and assessment at all time points was not required, as long as a pain score was performed by both Jr and Sr at the same time point. Three pain scores were excluded because no paired score was performed (i.e., score performed by Jr but not Sr, or vice versa). Twenty-four pain scores were obtained (12 Jr and 12 Sr) for comparison (Supplementary Table S1). At T_1_, senior pain scores were significantly different from junior pain scores (5.56 ± 0.54 vs. 3.22 ± 0.62, *p* = 0.005), with the senior evaluator obtaining greater scores than the junior (Figure 2). There was no significant difference between groups at T_0_ and T_2_.

To ensure accuracy and to limit the evaluator factor, only scores obtained from the senior evaluator (Sr) were used for Study 2.

### 3.3. Study 2—Analgesic Effect of NSAIDs According to the Pain Score

Of the 36 horses that were selected in Study 2, further exclusions were necessary due to incomplete pain evaluations, most likely due to lack of availability from the evaluator at T_2_ (*n* = 4, including 3 ketoprofen and 1 meloxicam), early post-operative colic episode (*n* = 1 ketoprofen) or an isolated episode of hyperthermia (*n* = 1 ketoprofen) requiring additional analgesic administration.

**Figure 3:**
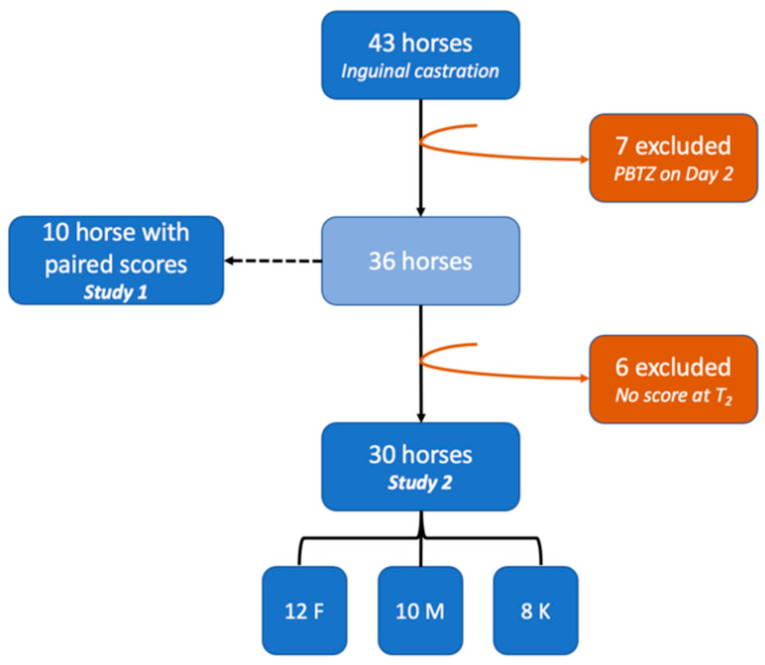
Flow diagram of group designation of horses after exclusion. PBTZ: phenylbutazone; F: flunixin meglumine; M: meloxicam; K: ketoprofen.

Complete data on thirty horses were finally used for statistical analysis (Figure 3). The mean age was 6.2 years (range 1.5–20 years), and represented breeds included French Warmbloods (12/30), mixed-breed leisure horses (7/30), Lusitanians (3/30), Spanish Purebred (2/30), one Arabian, one Irish Cob, one pony, one French Trotter, one Thoroughbred and one French chaser.

Ten horses were diagnosed with unilateral inguinal cryptorchidism which did not lead to a modification in surgical approach. There was no time–cryptorchidism interaction, and no effect of cryptorchidism on pain scores or NSAID assigned, allowing these horses to be included in further statistical analysis.

A total of 12 horses received flunixin meglumine, 10 meloxicam and 8 ketoprofen. There was no significant age difference between the three groups.

A wide range of delays was observed between the time of NSAID administration and pain score assessment, with a T_1_ ranging from 9 to 12.5 h (mean 10.5 h) and a T_2_ ranging from 1 to 9.5 h (mean 3.1 h). However, no statistically significant effect of delay on pain score was found at any time.

The evolution of pain scores with time was assessed for each NSAID, and mean pain scores are available in Table 2 (individual data available in Supplementary Table S2). With flunixin, the mean pain score at T_1_ was significantly different than at T_0_ (*p* < 0.001) and T_2_ (*p* < 0.001), with pain scores 3.5 times greater than at T_0_ and 2.2 times greater than at T_2_ (Table 3). The mean pain score at T_2_ was only mildly significantly different than at T_0_ (*p* = 0.0467). With meloxicam, the mean pain score at T_1_ was significantly different than at T_0_ (*p* < 0.001) and T_2_ (*p* < 0.0325), with pain scores 3.5 times greater than at T_0_ and 1.7 times greater than at T_2_. The mean pain score at T_2_ was significantly different than at T_0_ (*p* = 0.0217). With ketoprofen, the mean pain score at T_1_ and T_2_ were significantly different than at T_0_ (*p* < 0.0001 and *p* < 0.000514, respectively), with pain scores 3.75 times greater at T_1_ than at T_0_ and 2.75 times greater at T_2_ than at T_0_. There was no significant difference in pain scores between T_1_ and T_2_ with ketoprofen.

**Figure 4:**
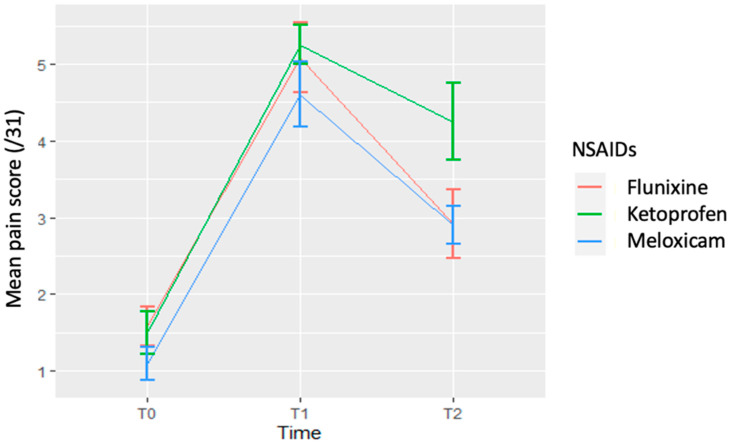
Mean pain scores depending on time and the administered NSAID. Data are reported as the mean and SEM.

When taking into account the simultaneous effect of time and NSAID on pain scores, there was no time–NSAID interaction. When comparing the mean pain scores of each NSAID without taking time into account, there was no significant effect of NSAIDs on pain scores, even though a less marked decrease in pain score was observed at T_2_ with ketoprofen than with flunixin and meloxicam. When comparing mean pain scores with time without taking NSAIDs into account, there was a significant time effect, with pain scores at T_1_ being significantly greater than at T_0_ and T_2_ (*p* < 0.0001 and *p* < 0.000126, respectively) (Figure 4).

## 4. Discussion

Non-steroidal anti-inflammatory drugs are one of the most commonly used drugs in horses for the management of inflammation and pain of myo-arthro-skeletal or visceral origins, with flunixin meglumine traditionally being the most used NSAID for visceral pain [1,14,15]. However, this traditional, non-selective anti-inflammatory drug inhibits both COX-1 and COX-2 isoforms and has been associated with numerous side effects, including gastrointestinal ulceration and delayed mucosal healing, and renal toxicity [3,4,5,15,16,17]. While COX-1 is expressed constitutively and mediates the homeostasis of gut barrier function, COX-2 is induced in pro-inflammatory states, and the use of COX-2-specific NSAIDs is warranted in order to reduce the risk of complications [18]. Meloxicam is a preferential COX-2 NSAID, with a COX-1:COX-2-selective half maximal inhibitory concentration (IC_50_) ratio of 3.8 (vs a ratio of 1 for flunixin), allowing sufficient activity of COX-1 required to repair the enteric mucosa, while preventing pain and inflammation due to COX-2 activity [17,19,20,21,22]. Ketoprofen is a nonselective NSAID, and PK/PD studies of ketoprofen in horses are scarce and conflicting. A study found lower ulcerogenic and renal effects of ketoprofen than that of phenylbutazone and flunixin, making ketoprofen an interesting alternative to flunixin for the management of visceral pain [3]. Other studies have found an IC_50_ ratio ranging from 0.48 to 2 depending on the methods and enantiomers evaluated, which could indicate a greater potency of ketoprofen for the inhibition of COX-1, and thus potential toxicity [23,24,25].

It is common belief that traditional non-specific NSAIDs are superior to COX-2-selective NSAIDs for pain management [1]. However, results from previous studies remain conflicting. A panel of specialists reported with moderate certainty that flunixin and firocoxib provided more effective analgesia than meloxicam or phenylbutazone in the case of colic [2]. Flunixin was found to provide superior analgesia than meloxicam in the post-operative period after strangulating small intestinal lesions, despite increased dosing frequencies of meloxicam [2,26]. Another study reported no difference in pain scores between meloxicam and flunixin in an experimental model of colic [21]. Some studies reported a lower analgesic effect of meloxicam than flunixin, however, clinicians were not blinded to treatment at the time of pain assessment [26]. In the current study, there was no difference in analgesic efficacy observed between flunixin, meloxicam and ketoprofen following inguinal castration, with pain scores returning close to normal at the same rate (within 24 h post-operatively) with flunixin and meloxicam. These findings are in agreement with a recent study evaluating the analgesic effect of these drugs following scrotal castration and supports the fact that meloxicam significantly reduces post-surgical pain and inflammation in horses following castration [27]. Equivalent levels of COX-2 inhibition were found between COX-2-selective (meloxicam or firocoxib) and nonselective (flunixin, phenylbutazone) anti-inflammatories, supporting a similar analgesic efficacy for meloxicam, firocoxib, phenylbutazone and flunixin [28].

Studies evaluating the analgesic effects of ketoprofen on visceral pain are scarce. While ketoprofen was found to not provide equipotent analgesia to phenylbutazone for the treatment of severe musculoskeletal pain, a single field study showed some analgesic effects of ketoprofen in horses with colic, but no comparison to other molecules was made, precluding from drawing conclusions [22,23,29]. In the current study, while no statistically significant difference was found between flunixin, meloxicam and ketoprofen, the latter showed a lower decrease in pain score 24 h post-operatively, which might suggest a slower or less efficacious analgesic effect of ketoprofen. One horse treated with ketoprofen presented with colic signs following surgery, which resolved after administration of flunixin meglumine. The horse was then pulled out of the study. Whether resolution of colic signs would have been observed with an additional administration of ketoprofen is unknown. Therapeutic decisions were made at the clinician’s discretion and based on personal experience.

NSAIDs act as analgesics by inhibiting COX and preventing the production of prostaglandins, in addition to a recently described central mechanism of action at the level of the spinal cord, unrelated to COX inhibition [22]. The analgesic effects of NSAIDs have a more rapid onset and shorter duration of action than the anti-inflammatory action. While the onset of the anti-inflammatory action of flunixin is within 2 h, with a peak response between 12 and 16 h and a duration of action of 36 h, ketoprofen reaches a peak response 4 h after administration and lasts for 24 h [22]. Other studies have described a more rapid and longer effect of flunixin than ketoprofen, while PK/PD of flunixin, meloxicam and phenylbutazone were similar [22,28,30,31,32]. In addition, ketoprofen was suggested to have altered plasma pharmacokinetics in the face of inflammation in peripheral compartments [33,34,35]. NSAIDs are known to be more effective as analgesics when inflammation is a part of the pain process and when they are given before the onset of the inflammatory process. Furthermore, the time to onset and the duration of analgesia of NSAIDs do not correlate well with their anti-inflammatory properties [22]. However, most studies evaluated the anti-inflammatory effects rather than the analgesic effects of these drugs. In the current study, the peak analgesic response of each NSAID was not evaluated, but no significant difference in analgesic efficacy was found between the molecules. On the other hand, while not significant, ketoprofen led to a lower decrease of pain score 24 h post-operatively, suggesting different pharmacokinetics from flunixin meglumine and meloxicam. Further studies focussing on the analgesic dynamics of NSAIDs are warranted.

Firocoxib is a highly COX-2-specific NSAID, with a COX-1:COX-2 selective IC_50_ ratio of 200, and has been proven to have analgesic efficacy similar to flunixin without retarding mucosal recovery [2,3,36]. However, firocoxib was not included in the current study as no intravenous formulation was available at the time. In order to prove the non-inferiority of meloxicam and ketoprofen to phenylbutazone for the management of visceral pain, the latter could have been included in the study. However, numerous studies have shown significant side effects (gastrointestinal ulceration, renal toxicity, right dorsal colitis) with the use of phenylbutazone and a lower efficacy than flunixin against visceral pain [2]. In addition, no intravenous formulation of phenylbutazone registered for horses was available at the time.

Recent studies have found that COX-2 are also physiologically expressed in the central nervous system, kidneys, ovaries, placenta and bones. In addition, both COX-1 and COX-2 participate in the integrity of gastrointestinal mucosa and renal function [14,37,38,39]. Thus, while COX-2-selective NSAIDs were proven to cause less side effects to the gastrointestinal mucosa, one should not forget that COX-2 inhibition may lead to renal toxicity, especially in volume-depleted horses, and that the administration of COX-2-specific NSAIDs is not without a harmful effect [4]. In addition, in the current study, one horse receiving meloxicam developed colitis post-operatively. While a direct harmful effect of meloxicam following a single administration is unlikely, a causative effect could not be ruled out.

The PASPAS pain score was selected and has been previously used for the assessment of post-castration pain [6,13]. This pain assessment scale was developed for horses following colic laparotomy and was not influenced by anesthesia, and was thus considered adequate for the evaluation of visceral pain following inguinal castration. The PASPAS scale was reported to be reliable with low inter-observer variability. However, all observers were experienced veterinarians, and reliability of the score when assessed by veterinary students was unknown. In the current study, poor agreement was found between an experienced internal medicine clinician and a fourth-year veterinary student. Pain scores were significantly greater when established by the senior evaluator at the time of highest pain (T_1_). In one case, the horse was miscategorized as mildly painful by the student, while considered moderately painful by the senior. This horse was actually showing colic signs which the student most likely missed. While some subcategories of PASPAS showed good correlation with the total pain score index (general subjective assessment, postural behavior, response to food), some parameters were found to have no correlation with the total pain score index, including a reaction to palpation of the incisional area and colic behavior [13]. It is most likely that experience is required in order to obtain accurate subjective assessments of pain, which a veterinary student may lack. In the current study, scores were assessed by a fourth-year student out of a five-year course. The fourth year is the first year of clinical training, so the student was in the process of training to assess pain. Moreover, this particular student had experience with horses so should be familiar with abnormal behaviors. However, our findings are in agreement with previous publications showing a significant difference in pain scores between veterinary students and experienced anesthesiologists or veterinarians [9,10,40]. Another composite pain scale suggested no need for training before its use [40,41], however, while veterinary students were involved in pain assessment, no comparison between veterinary students and experienced veterinarians was performed. According to our findings, and previous publications, training seems necessary in order to accurately assess pain in horses, and the pain score used in the present study is not recommended for the assessment of pain by untrained veterinary students or owners.

In the current study, incisional edema was evaluated as a sign of inflammation and replaced palpation of the incisional area. In their study, Gobbi et al. found greater local edema with firocoxib and meloxicam than flunixin, in agreement with other studies supporting the low anti-edema activity of meloxicam [26,42]. The effect of each NSAID on subcategories of the pain score was not assessed in the current study, and despite the fact that there were no significant differences in pain scores between NSAIDs, the correlation between the degree of incisional edema and the total pain index warrants further investigation.

These findings are in agreement with a panel of experts highlighting the sparsity of publications about pain scores and the lack of gold standards for the quantification of pain in horses [2,40]. The reliability of pain scores is highly variable depending on studies, and physiological parameters have been found to be weakly associated with pain, while behavioral changes are often easier to evaluate and considered more pain-specific [43]. Furthermore, this makes it difficult to interpret publications using a wide variety of pain assessment scales for the evaluation of the analgesic effects of NSAIDs. A standardized, easy-to-use, composite pain score with good inter-observer reliability is required in order to accurately evaluate the efficacy of analgesic agents in horses.

### Limitations

Possible side effects of each NSAID were not evaluated. Repeat bloodwork, including assessment of renal function, gastroscopy and abdominal ultrasounds could have been performed, but were considered unpractical and not financially sustainable considering the electiveness of the surgical intervention and the financial implication for horses’ owners. Horses exhibiting severe pain post-operatively were excluded from the study, which may have biased the statistical analysis. However, these horses required additional pain management which would have affected future pain scores, and withholding analgesic medication until the next scheduled dose would have been unethical.

All horses received local anesthesia with lidocaine pre-operatively. Studies have shown significant decreases in pain scores following the local administration of lidocaine or mepivicaine before castration [2,7]. Lower levels of post-operative pain may have partially masked the different analgesic efficacies between NSAIDs. In their study, Graubner et al. found no influence of anesthesia on PASPAS when assessed 8 h after recovery [13]. However, another study suggests that while some pain-related behaviors were not influenced by anesthesia, analgesia, or time of day, the latter was a confounding factor for other behaviors (walk, rest standing still, look out window, rest pelvic limb) included in the subjective assessment part of our modified score [44]. In the current study, the time from recovery to pain score assessment was variable, and while no effect of time on pain scores was found, some remnant effects of anesthesia when assessed less than 8 h post-recovery could not be ruled out.

All horses received anti-edematous medication, which contained dexamethasone and thus had anti-inflammatory effects. However, as all horses received the same medication, the effect on NSAID analgesic effects, pain scores and assessment of incisional edema was considered minimal. Finally, the small sample size warrants cautious interpretation of the results. It was the author’s choice not to include horses admitted for scrotal castration, as it possibly would have led to various levels of scrotal edema and different levels of pain.

## 5. Conclusions

Inguinal castration most often led to only minimal pain, which was similarly managed with meloxicam, ketoprofen and flunixin. This study warrants the use of meloxicam and ketoprofen with a similar level of analgesia than flunixin for the management of mild visceral pain. The PASPAS pain score was not reliable for pain assessment by a veterinary student, and highlights the need for of a gold standard for assessment of visceral pain in horses.

## Figures and Tables

**Table 1:** Composite pain score adapted from Graubner et al. [4]. Total score (out of 31): score ≤ 7: mid pain; score = 8–14: moderate pain; score > 14: severe pain.

	Date	/
	Time of last treatment	**:**
	Time of evaluation	**:**
**Category**	**Manifestation(s)**	**Score**
General subjective assessment	No sign of pain	0
		1
		2
		3
	Sign of severe pain	4
Heart rate (beats/min)	<40	0
	40–49	1
	50–59	2
	>60	3
Respiratory rate (breaths/min)	<20	0
	20–30	2
	>30	4
Postural behavior	No reaction to vocal and environmental stimuli	1
	Standing still	1
	Arched back, tucked-up belly	1
Interactive behavior	Interested	0
	Looks at observer	1
	Moves away	2
	Does not move	3
Response to food	Strong appetite	0
	Appetite but wearing a muzzle	1
	Little appetite	2
	No appetite at all	4
Colic behavior	No colic signs shown	0
	Paws intermittently	1
	Paws and lies down	2
	Looks at the flank, paws frequently	3
	Rolls, kicks against the abdomen	5
	Keeps throwing himself down	6
Stimulation of muscle Th17-L1	No reaction	0
	Hardened muscles, reaction shown	1
Aspect of wound	No oedema	0
	Minimal oedema, hardly visible	1
	Moderate oedema (<size of tangerine)	2
	Marked oedema (>size of tangerine)	3
TOTAL		

**Table 2:** Mean ± standard deviation of pain scores, depending on time and NSAIDs. Pain scores were assessed before NSAID administration (T_0_), after recovery from anesthesia (T_1_) and after the second NSAID administration (T_2_).

	T_0_		T_1_		T_2_	
	**Mean** ± **SD**	**(Min; Max)**	**Mean** ± **SD**	**(Min; Max)**	**Mean** ± **SD**	**(Min; Max)**
Flunixin	1.58 ± 1.38	(0; 4)	5.08 ± 2.50	(0; 7)	2.92 ± 2.423	(0; 6)
Meloxicam	1.10 ± 1.20	(0; 3)	4.60 ± 2.32	(0; 8)	2.90 ± 1.37	(0; 3)
Ketoprofen	1.50 ± 1.51	(0; 4)	5.25 ± 1.39	(3; 6)	4.25 ± 2.76	(0; 7)

**Table 3:** The effect of time, NSAIDs and both on mean pain scores. Pain scores were assessed before NSAID administration (T_0_), after recovery from anesthesia (T_1_) and after the second NSAID administration (T_2_). The statistical results of multiple comparisons of means using post-hoc Tukey tests are given.

	Mean’s Difference Estimate	Std.Error	*p* Value
Effect of time and NSAID on pain score			
Flunixin			
T_1_–T_0_	3.5000	0.5626	<0.001 *
T_2_–T_0_	1.3333	0.5626	0.0468 *
T_2_–T_1_	−2.1667	0.5626	<0.001 *
Meloxicam			
T_1_–T_0_	3.5000	0.6782	<0.001 *
T_2_–T_0_	1.8000	0.6782	0.0217 *
T_2_–T_1_	−1.7000	0.6782	0.0325 *
Ketoprofen			
T_1_–T_0_	3.7500	0.7278	<1 × 10^−4^ *
T_2_–T_0_	2.7500	0.7278	0.000514 *
T_2_–T_1_	−1.0000	0.7278	0.354615
Effect of time on pain score			
T_1_–T_0_	3.5667	0.3689	<0.0001 *
T_2_–T_0_	1.8667	0.3689	<0.0001 *
T_2_–T_1_	−1.7000	0.3689	<0.000126 *
Effect of NSAIDs on pain score			
K–F	0.4722	0.7228	0.790
M–F	−0.3278	0.6780	0.879
M–K	−0.8000	0.7511	0.535

* Significance set at *p* ≤ 0.05.

## Data Availability

The data presented in this study are available on request from the corresponding author. The data are not publicly available due to privacy.

## Supplementary Materials

The following supporting information can be downloaded at https://www.mdpi.com/article/10.3390/ani12040526/s1: Table S1: Individual data Study 1—Variability of pain score based on evaluator’s experience; Table S2: Individual data Study 2—Analgesic effect of NSAIDs according to the pain score.
