# Supplementary material for: Comparison of Flunixin Meglumine, Meloxicam and Ketoprofen on Mild Visceral Post-Operative Pain in Horses
Source: Animals (Basel). 2022 Feb 21;12(4):526. doi: 10.3390/ani12040526 (PMC8868280; doi:10.3390/ani12040526)
Supplement: Supplementary file 1 [file animals-12-00526-s001.zip › animals-1577621-supplementary.pdf]

Table S1: Individual data Study 1 - Variability of pain score based on evaluator's experience																																				
Case	Evaluator	NSAID	Time drug - score (hours)		Pain score assessment																											Pain score /31				
					Subjective assessment			HR			RR			Posture			Interaction			Appetite			Colic			Muscle stimulation			Wound							
			T1	T2	T0	T1	T2	T0	T1	T2	T0	T1	T2	T0	T1	T2	T0	T1	T2	T0	T1	T2	T0	T1	T2	T0	T1	T2	T0	T1	T2	T0	T1	ΔT1	T2	ΔT2
					/4	/4	/4	<40 = 0 ; 40-49 = 1 ; 50-89=2 ; >60 = 3			<20 = 0 ; 20-30 = 2 ; >30 = 4			/3	/3	/3	/3	/3	/3	/4	/4	/4	/6	/6	/6	/1	/1	/1	/3	/3	/3					
1	Sr	K	7,5		0	1		1	1		2	0		0	1		0	1		0	1		0	0		0	0		0	1		3	6	3		
1	Jr	K	7	0	0	2	0	1	2	1	2	2	2	0	1	0	0	1	0	0	1	0	0	0	0	0	0	0	0	0	0	3	9	6	3	0
2	Sr	M	12	3	0	1	0	0	0	0	0	0	2	0	0	0	0	0	0	0	0	0	0	0	0	0	0	0	0	1	1	0	2	2	3	3
2	Jr	M	10	8	0	0	1	0	0	1	0	0	0	0	0	0	0	0	0	0	0	0	0	0	0	0	0	0	0	0	0	0	0	0	2	2
3	Sr	K		3	0		1	2		0	0		0	0		0	0		0	0		0	0		0	0		0	0		1	2			2	0
3	Jr	K	9,5	0,5	0	1	0	2	1	1	0	0	0	0	0	0	0	1	0	0	1	0	0	0	0	0	0	0	0	1	2	2	5	3	3	1
4	Sr	M	11	9	0	2	1	0	1	1	0	2	0		0	0	0	0	0	0	1	0	0	0	0	0	0	0	0	2	1	0	8	8	3	3
4	Jr	M	9,5	0,5	0	0	0	1	1	0	0	0	0	0	0	0	0	0	0	0	1	0	0	0	0	0	0	0	0	1	1	1	3	2	1	0
5	Sr	F	11	1	0	2	1	0	1	0	0	0	0	0	1	0	0	0	0	0	0	0	0	0	0	0	1	0	0	2	1	0	7	7	2	2
5	Jr	F	8	1	0	1	0	1	0	0	0	0	0	0	0	0	0	1	0	0	1	0	0	0	0	0	0	0	0	2	2	1	5	4	2	1
6	Sr	K	9,5	3,5	0	2	0	0	1	1	0	0	0	0	0	0	0	0	0	0	0	0	0	0	0	0	1	0	0	2	1	0	6	6	2	2
6	Jr	K	9	1	0	0	0	0	1	0	0	0	0	0	0	1	0	0	0	0	0	0	0	0	0	0	0	0	0	1	2	0	2	2	3	3
7	Sr	F	11	1,5	0	1	0	0	0	0	0	0	0	0	0	0	0	0	0	0	0	0	0	0	0	0	0	0	0	2	1	0	3	3	1	1
7	Jr	F	7	1	0	1	0	0	0	0	0	0	0	0	0	0	0	0	0	0	0	0	0	0	0	0	0	0	0	1	1	0	2	2	1	1
8	Sr	K	8		0	2		0	0		0	0		0	1		0	0		0	1		0	1		1	1		0	2		1	8	7		
8	Jr	K	9	1	0	1	0	0	0	0	2	0	0	0	0	0	0	0	0	0	0	0	0	1	0	0	0	1	0	2	3	2	4	2	4	2
9	Sr	K	12	2	0	1	0	0	0	0	0	0	0	0	0	0	0	0	0	0	0	0	0	0	0	0	0	0	0	2	2	0	3	3	2	2
9	Jr	K	10	0,5	0	0	0	0	0	0	0	0	0	0	0	0	0	0	0	0	1	0	0	0	0	0	0	0	0	0	2	0	1	1	2	2
10	Sr	F	10	9,5	0	1	0	0	2	0	2	0	0	0	0	0	0	0	0	0	1	0	0	0	0	0	1	0	0	2	2	2	7	5	2	0
10	Jr	F	10	1,5	0	0	0	0	1	0	0	0	0	0	0	0	0	0	0	0	0	0	0	0	0	0	0	0	0	2	2	0	3	3	2	2

Sr: Senior; Jr: Junior; K: Ketoprofen; M: Meloxicam; F: Flunixin; T: time of pain assessment; ΔT1 = T1 – T0; ΔT2 = T2 – T0

Table S2: Individual data Study 2 - Analgesic effect of NSAIDs according to the pain score

Case	Evaluator	NSAID	Time drug - score (hours)		Pain score assessment																															Pain score /31				
					Subjective assessment			HR			RR			Posture			Interaction			Appetite			Colic			Muscle stimulation			Wound			T0	T1	ΔT1	T2	ΔT2
			T1	T2	T0	T1	T2	T0	T1	T2	T0	T1	T2	T0	T1	T2	T0	T1	T2	T0	T1	T2	T0	T1	T2	T0	T1	T2	T0	T1	T2					
					/4	/4	/4	<40 = 0 ; 40-49 = 1 ; 50-89=2 ; >60 = 3			<20 = 0 ; 20-30 = 2 ; >30 = 4			/3	/3	/3	/3	/3	/3	/4	/4	/4	/6	/6	/6	/1	/1	/1	/3	/3	/3					
1	Sr 1	K	13	1,5	0	2	0	0	0	0	2	0	2	0	0	0	0	0	0	0	2	0	0	0	0	1	1	0	0	2	2	3	7	4	4	1
2	Sr 1	K	9	3	0	1	2	0	1	1	0	0	0	0	1	0	0	0	0	0	0	0	0	0	1	0	0	0	0	2	3	0	5	5	7	7
3	Sr 1	M	9	3	0	1	0	1	1	0	0	0	0	0	1	0	0	0	0	0	0	0	0	0	0	0	1	0	0	1	0	1	5	4	0	-1
4	Sr 1	K	12	2	0	1	0	0	0	0	0	0	0	0	0	0	0	0	0	0	0	0	0	0	0	0	0	0	0	2	2	0	3	3	2	2
5	Sr 1	M	12	3	0	1	0	0	0	0	0	0	2	0	0	0	0	0	0	0	0	0	0	0	0	0	0	0	0	1	1	0	2	2	3	3
6	Sr 1	K	9	3	0	1	1	2	1	0	0	0	0	0	0	0	0	1	0	0	1	0	0	0	0	0	0	0	0	1	1	2	5	3	2	0
7	Sr 1	M	11	9	0	2	1	0	1	1	0	2	0		0	0	0	0	0	0	1	0	0	0	0	0	0	0	0	2	1	0	8	8	3	3
8	Sr 1	F	11	1,5	0	1	0	0	0	0	0	0	0	0	0	0	0	0	0	0	0	0	0	0	0	0	0	0	0	2	1	0	3	3	1	1
9	Sr 1	F	11	1	0	2	1	0	1	0	0	0	0	0	1	0	0	0	0	0	0	0	0	0	0	0	1	0	0	2	1	0	7	7	2	2
10	Sr 2	F	9	6,5	0	0	0	0	0	0	0	0	0	0	0	0	0	0	0	0	0	0	0	0	0	0	0	1	0	0	0	0	0	0	1	1
11	Sr 1	M	10	1	0	1	0	1	1	1	2	0	2	0	0	0	0	0	0	0	0	0	0	0	0	0	0	0	0	1	1	3	3	0	4	1
12	Sr 2	M	13	1	0	0	0	0	0	0	0	0	2	1	0	0	0	0	0	0	0	0	0	0	0	0	1	0	0	0	1	1	1	0	3	2
13	Sr 1	M	10	1,5	0	1	1	0	0	0	0	0	0	0	0	0	0	0	0	0	0	0	0	0	0	0	0	0	0	2	1	0	3	3	2	2
14	Sr 1	F	10	2	0	2	2	1	2	2	2	4	4	0	0	0	0	0	0	0	0	0	0	0	0	0	0	0	0	1	1	3	9	6	9	6
15	Sr 1	F	11	3,5	0	2	1	1	1	1	0	0	0	0	1	0	0	1	0	0	0	0	0	0	0	0	0	0	0	1	1	1	6	5	3	2
16	Sr 2	K	11	2	0	1	0	0	0	0	0	0	0	0	1	0	1	1	1	0	0	0	0	0	0	0	1	1	0	0	0	1	4	3	2	1
17	Sr 2	K	10	2	0	1	2	1	0	0	0	0	0	0	0	0	1	0	1	0	1	0	0	0	0	0	1	1	0	2	2	2	5	3	6	4
18	Sr 2	F	11	1	0	1	1	1	0	0	0	2	2	0	0	0	1	0	0	0	1	0	0	0	0	1	1	0	0	0	0	3	5	2	3	0
19	Sr 2	F	11	7	0	0	1	0	0	0	0	2	0	0	0	0	0	0	0	0	1	0	0	0	0	1	0	0	0	1	0	1	4	3	1	0
20	Sr 1	M	-	-	0	1	0	0	1	1	0	2	2	0	1	0	0	0	0	1	0	0	0	0	0	0	0	0	0	2	1	1	7	6	4	3
21	Sr 2	M	9,5	1,5	0	1	0	0	0	0	0	2	0	0	0	0	0	0	0	0	0	0	0	0	0	0	0	0	0	2	2	0	5	5	2	2
22	Sr 1	F	10	9,5	0	1	0	0	2	0	2	0	0	0	0	0	0	0	0	0	1	0	0	0	0	0	1	0	0	2	2	2	7	5	2	0

23	Sr 1	F	10	1	0	0	0	1	2	1	0	0	0	0	0	0	0	0	0	0	1	0	0	0	0	0	0	0	0	1	0	1	4	3	1	0
24	Sr 1	F	12	2,5	0	1	1	0	0	1	0	0	0	0	0	0	0	1	0	1	0	0	0	0	0	0	0	0	0	1	2	1	3	2	4	3
25	Sr 1	K	12	-	0	1	3	1	1	1	2	2	0	0	0	0	0	1	1	1	1	2	0	0	0	0	1	1	0	0	1	4	7	3	9	5
26	Sr 1	K	9,5	3,5	0	2	0	0	1	1	0	0	0	0	0	0	0	0	0	0	0	0	0	0	0	0	1	0	0	2	1	0	6	6	2	2
27	Sr 1	M	9,5	3	0	1	0	1	1	1	2	2	2	0	0	0	0	0	0	0	1	0	0	0	0	0	0	0	0	2	2	3	7	4	5	2
28	Sr 2	F	11	1	0	1	0	1	0	0	2	2	0	0	0	0	0	0	0	0	1	0	0	0	0	0	0	0	0	1	2	3	5	2	2	-1
29	Sr 2	F	11	1,5	0	3	2	1	0	0	2	0	0	0	2	1	1	1	1	0	1	1	0	0	0	0	0	0	0	1	1	4	8	4	6	2
30	Sr 2	M	11	8,5	0	1	0	0	0	0	2	0	0	0	1	0	0	0	0	0	1	0	0	0	0	0	0	1	0	2	2	2	5	3	3	1

Sr 1: AL; Sr 2: LL; K: Ketoprofen; M: Meloxicam; F: Flunixin; T: time of pain assessment; ΔT1 = T1 – T0; ΔT2 = T2 – T0
